# A Model of the Current Geographic Distribution and Predictions of Future Range Shifts of *Lentinula edodes* in China Under Multiple Climate Change Scenarios

**DOI:** 10.3390/jof11100730

**Published:** 2025-10-10

**Authors:** Wei-Jun Li, Rui-Heng Yang, Ting Guo, Sheng-Jin Wu, Yu Li, Da-Peng Bao

**Affiliations:** 1 Engineering Research Center of Chinese Ministry of Education for Edible and Medicinal Fungi, Jilin Agricultural University, Changchun 130118, China; liweijun6622@163.com; 2 Key Laboratory of Edible Fungal Resources and Utilization (South), Ministry of Agriculture, National Engineering Research Center of Edible Fungi, Key Laboratory of Agricultural Genetics and Breeding of Shanghai, Institute of Edible Fungi, Shanghai Academy of Agricultural Sciences, Shanghai 201403, China; 3 Institute of Microbiology, Guangxi Academy of Agricultural Sciences, Nanning 530007, China

**Keywords:** fungal biogeography, fungal conservation, MaxEnt, population changes, suitable habitat

## Abstract

Due to its ecological functions, huge economic benefits, and excellent nutritional and physiological activities, *Lentinula edodes* is a very popular edible fungus in Asia, especially in China. Changes in the distribution and population of wild *L. edodes* play an important role in conservation, variety improvements, and breeding. This investigation detected wild *L. edodes* in 28 provinces and municipalities in China, encompassing approximately 300 regions and natural reserves. MaxEnt analysis of 53 effective distribution locations indicated that host plants, Bio19 (precipitation in the coldest quarter), Bio10 (mean temperature of the warmest quarter), and Bio17 (precipitation in the driest quarter) made the most critical contributions to this model. The areas of suitable and highly suitable habitats were 55.386 × 10^4^ km^2^ and 88.493 × 10^4^ km^2^, respectively. Under four climate change scenarios, the *L. edodes* distribution was predicted to decrease and the suitable habitat area shifted to the north and west of China. The decrease in highly suitable habitat area ranged from 21.155% in the 2070s under the ssp1-2.6 scenario to 90.522% in the 2050s under the ssp3-7.5 scenario. This sharp reduction in habitat areas suggests that we should take measures to prevent the deterioration of the environment and climate and thus to ensure the survival of *L. edodes*.

## 1. Introduction

*Lentinula* (Agaricales, Omphalotaceae), characterized as white rot wood decayers, are widely distributed fungi globally, with the exception of Europe and Antarctica [1,2]. They are able to degrade all polymers from lignocellulosic biomass, including cellulose, hemicelluloses and lignin, using enzymatic systems [3,4,5]. This genus (*Lentinula*) includes *L. edodes* (Berk.) Pegler, which is one of the most widely cultivated edible mushrooms [6]. Due to its high content of polysaccharides, terpenoids, sterols, and lipids, *L. edodes* has demonstrated immunomodulation, antitumor, antioxidant, and antiviral activities [7,8,9]. It is one of the most important and popular edible mushrooms in China, Japan and Korea [6]. *L. edodes* has been artificially cultivated for more than 800 years, originating in China in Qingyuan, Zhejiang province, placing it as the flagship mushroom of China [1,10]. The total production of *L. edodes* reached 10 million tons in 2023, accounting for 90% of the total mushroom production.

Investigations have revealed that in China, *L. edodes* is distributed from east to west and from south to north, covering 21 provinces and regions [11]. The genetic diversity of *L. edodes* in China is much higher than that of any other region [11,12]. Based on RAPD, AFLP, SSR and genome sequencing results [5,12,13,14,15,16,17], it has been found that in China, *L. edodes* is clustered into three distinct subgroups according to geographical distribution. Group A includes wild strains from Yunnan and Sichuan provinces in southwest China; group B contains wild strains from central China; and the strains in group C mainly include strains from northeast China and cultivated strains. All the results indicate that wild resources have an abundant genetic diversity while cultivated strains are genetically similar [5,16,17]. The abundant genotypes in wild *L. edodes* may play an important role in variety improvement and breeding. Therefore, the protection of wild *L. edodes* is of great importance.

It is known that the climate greatly impacts the structure and function of terrestrial ecosystems on both the temporal and spatial scales [18] and affects the growth and reproduction of species [19]. Therefore, potential changes in climate and the environment may alter biodiversity and habitat distributions and cause habitat fragmentation, all of which are potential threats to endangered animals, plants, and fungi [19]. Global warming is an indisputable fact, and in response to this change, the distributions of some plant, animal and fungus species have shifted towards the poles or to higher elevations [20,21]. If shifts in distribution ranges continue indefinitely for animals, plants and fungi, range expansion or contraction may occur, or even extinction for some species [22]. Predicting current geographic distributions and future range shifts is important for providing advice regarding the protection and sustainable utilization of resources [23].

For animals and plants, species distribution models (SDMs) are widely used for predicting the geographic distribution based on currently their known distribution in association with various environmental variables from these locations [24,25,26,27,28,29,30,31]. However, there are few studies detailing species distribution models for fungi. Over the past two decades, the geographic distributions of *Cordyceps cicadae* Miq. [32], *Fusarium oxysporum* Schltdl. [33], *Tricholoma matsutake* (S. Ito & S. Imai) Singer [34], *Ophiocordyceps sinensis* (Berk.) G.H. Sung, J.M. Sung, Hywel-Jones & Spatafora [28,35,36], *Sanghuangporus* (including ten species of *Sanghuangporus*) [37], *Sclerotinia sclerotiorum* (Lib.) de Bary [38] and *Monilinia fructicola* (G. Winter) Honey [39] have been predicted. The results reveal that species distribution models (e.g., MaxEnt) are useful tools to predict the distribution ranges of these fungi and provide important ecological information for their utilization and conservation. Although *L. edodes* is classified as Least Concern (LC) in the IUCN Red List of Fungi (https://www.mee.gov.cn/xxgk2018/xxgk/xxgk01/201805/t20180524_629586.html (accessed on 24 May 2018)), empirical evidence shows that the quantity of its wild resources has declined, and it has become more difficult to collect new sporophores in its existing distribution areas. Specifically, local communities worldwide perceive a significant reduction in the abundance of wild edible plants and mushrooms, with *L. edodes* and other mushroom species included in this trend [39]. Additionally, this observation aligns with our own experiences during field collection. All the results suggest that the distribution of wild *L. edodes* is shifting; however, whether the distribution of *L. edodes* is decreasing in response to climate change requires further exploration through robust data and comprehensive analyses.

In this study, the distribution and range shifts of *L. edodes* in China were predicted using a MaxEnt model based on a comprehensive dataset. The aims of this study are as follows: (1) to investigate whether and how host plants and climate change will affect the distribution of *L. edodes* and (2) to predict the potential range shifts of this fungus in response to climate change in the medium term of about 50–70 years. The results presented here could provide important ecological information and facilitate the utilization and conservation of this fungus.

## 2. Materials and Methods

### 2.1. Occurrence Data

Three methods were used to collect occurrence data: The first method was a literature survey, in which “*Lentinus edodes*”, “*Lentinula edodes*”, “Shiitake” and “Xianggu” were searched on the Web of Science, Pubmed, CNKI (www.cnki.net), and some other databases. Studies with clear collection sites and wild strains were reviewed. The second method was data collection using herbariums, and the third method was based on field collection experiments. Only samples with detailed latitude and longitude data were used in the MaxEnt models. Subsequently, a spatial filtering procedure was implemented to minimize spatial autocorrelation: when the distance between two occurrence records was less than 10 km, only one representative point was retained. This approach improved the representativeness of the data in relation to actual species distribution patterns.

### 2.2. Environmental Variables

To model the present geographic distribution of *L. edodes*, a total of 19 bioclimatic indicators, along with the corresponding elevation data, were downloaded from the WorldClim version 2.1 database and utilized for subsequent analysis. For climate data from 1970–2000, the spatial resolution of these environmental variables is 30 s (ca. 1 km).

Four different future emission scenarios were proposed for the four 20-year periods to the year 2100, viz., the 2030s (2021–2040), the 2050s (2041–2060), the 2070s (2061–2080), and the 2090s (2081–2100), each corresponding to four Shared Socio-economic Pathways (SSPs) in the CMIP6 model of IPCC AR6 (viz., SSP1-2.6, SSP2-4.5, SSP3-7.0, and SSP5-8.5). For the current climate (baseline), we used historical climate data from the WorldClim database, with SSPs spanning five different global climate scenarios, viz., SSP1, corresponding to Sustainability—Taking the Green Road (low challenges to mitigation and adaptation); SSP2, corresponding to the Middle of the Road scenario (medium challenges to mitigation and adaptation); SSP3, corresponding to Regional Rivalry—A Rocky Road (high challenges to mitigation and adaptation); SSP4, corresponding to Inequality—A Road Divided (low challenges to mitigation, high challenges to adaptation); and SSP5, corresponding to Fossil-fueled Development—Taking the Highway (high challenges to mitigation, low challenges to adaptation). In order to model the future geographic distribution of *L. edodes*, bioclimatic indicators from the BCC-CSM2-MR general circulation model—at a spatial resolution of 30″ (approximately 1 km^2^)—were downloaded. Notably, the bioclimatic indicators corresponding to SSP4 were unavailable. In addition to the commonly used bioclimatic variables and elevation data, host plants are recognized as critical factors restricting *L. edodes* growth. Therefore, the distribution of each host plant genus (*Carpinus*, *Castanea*, *Castanopsis*, *Coriaria*, *Lithocarpus*, *Quercus*) associated with *L. edodes* was retrieved from the Global Biodiversity Information Facility (GBIF https://www.gbif.org/, accessed on 23 October 2023) as a main variable. Using ArcGIS, the number of host plant occurrences at each coordinate was converted to 30″ (≈1 km^2^) raster data to model the current geographic distribution. To test the importance of host plants in the future geographic distribution, the current models were also run excluding host plants from the environmental variables. When modeling the future geographic distribution, the elevation variable was assumed to be constant over all analyzed time periods, while the distribution of host plants (6 genera) [40] was predicted with the same method under multiple scenarios. The predicted niche suitability index of the host plants (in percentage) was converted to raster data using ArcGIS at a spatial resolution of 30 s (approximately 1 km^2^). If more than one host plant genera was present in a single raster, the highest niche suitability index from these genera was selected to represent this raster.

### 2.3. Model Evaluation

The distribution of *L. edodes* was predicted using MaxEnt (vision 3.4.4). Of all known occurrence records, 75% were randomly selected as training data, with the remaining 25% serving as the test set [32,37]. The maximum number of algorithm iterations was set to 1000. The entire process was repeated 10 times to minimize variability arising from random sampling. A Jackknife test assessed the relative importance of environmental variables for the potential distribution. All other parameters were set to default values.

To evaluate the accuracy of the current geographic distribution modeled by the MaxEnt model and thus to assess the discriminatory power of the species distribution model [41], the widely used Area Under the Receiver Operator Characteristic Curve (AUC) statistic was employed. Model performance was classified as “good” and “excellent” when the AUC value exceeded 0.8 and 0.9, respectively. Niche suitability levels were defined using the following criteria: 0–25%, unsuitable; 25–50%, low suitability; 50–75%, moderate suitability; and 75–100%, high suitability [42]. Response curves of the key environmental variables for the distribution models were generated. Furthermore, using ArcGIS 10.7, we calculated the centroid migration distances (coordinates and displacement) and changes in area across the four suitability levels over the four time periods under the four scenarios, comparing these to the current potential distribution.

## 3. Results

### 3.1. Geographical Distribution of Wild L. edodes in China

**Figure 1:**
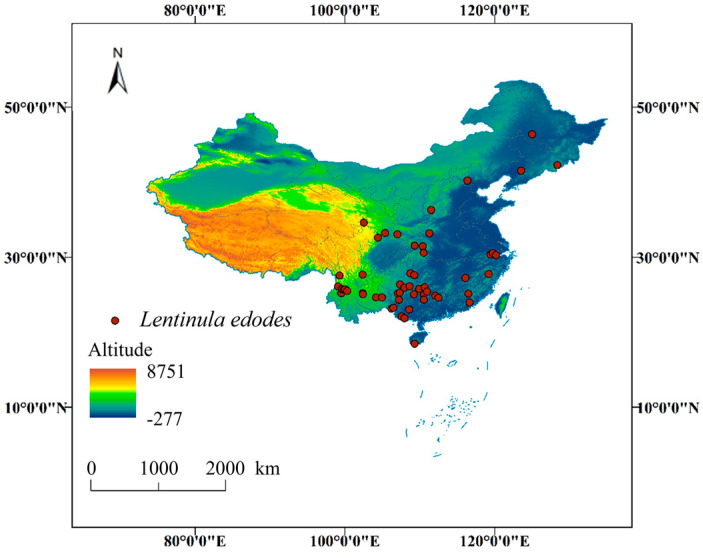
Occurrence records of *L. edodes* in China, used for MaxEnt analysis.

At present, wild *L. edodes* has been recorded in 28 provinces and municipalities in China, encompassing approximately 300 regions and natural reserves (Table S1). These areas extend from the south to the north and from the east to the west. These provinces are Anhui, Fujian, Gansu, Guangdong, Guangxi, Guizhou, Hainan, Hebei, Henan, Heilongjiang, Hubei, Hunan, Jilin, Jiangsu, Jiangxi, Liaoning, Qinghai, Shandong, Shanxi, Shaanxi, Sichuan, Taiwan, Tibet, Xinjiang, Yunnan, Zhejiang, Chongqing, and Hainan. Yunnan Province has the largest number of records at 54, followed by Sichuan (37), Guizhou (36), Jiangxi (25), Guangxi (20), and Fujian (19); these provinces are mainly located in southern China. There are no more than five records in 11 provinces, 7 of which are in the north China. For Beijing, Jiangsu, Qinghai, Shandong, Xinjiang and Hainan, there is no specific collection location or only one collection location. Unfortunately, there are no records in Macao, Ningxia, Shanghai, Tianjin, Hong Kong and other places. Only occurrence records with detailed geographical coordinates and inter-point distances exceeding 10 km were retained. A total of 54 effective distribution points were ultimately selected (Table S2, Figure 1). The filtered distribution data were saved in CSV format to facilitate subsequent analyses.

### 3.2. Model Accuracy

**Figure 2:**
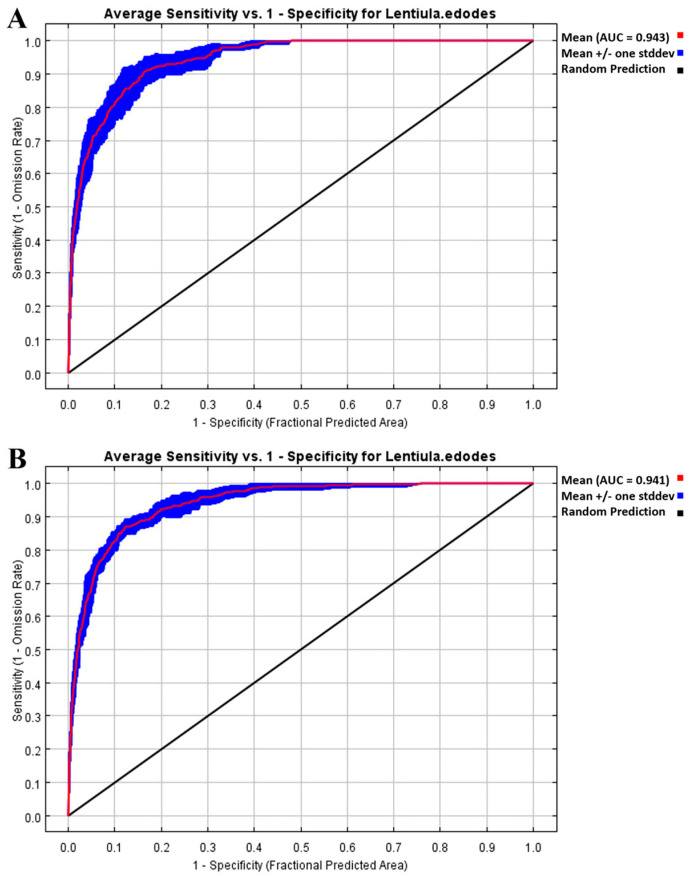
Receiver Operating Characteristic (ROC) curves for testing the performance of MaxEnt model in predicting *L. edodes* distribution under different plant host conditions: (**A**) without plant hosts; (**B**) with six plant hosts.

The AUC value of the *L. edodes* distribution model was 0.943 when the host plant variable included all six plants, while it was 0.941 when the host plant variable was excluded (Figure 2). Theoretically, when the AUC exceeds 0.9, the model performs well. Both AUC values obtained in this study surpass this threshold, indicating that the MaxEnt model performed well in predicting *L. edodes* distribution under both scenarios. Furthermore, as depicted in Figure 2, the mean ROC curves (along with their standard deviation ranges) for both models lie substantially above the diagonal “Random Prediction” line—a pattern that further confirms the models do not rely on random guessing and possess reliable predictive power.

### 3.3. Main Environmental Variables

Host plants were the key environmental variables influencing the distribution of *L. edodes*, with a contribution exceeding 51.5%. In the model including the six host plants, Bio19 (precipitation in the coldest quarter), Bio10 (mean temperature of the warmest quarter), and Bio17 (precipitation in the driest quarter) contributed to 9.7%, 5.7%, and 5.6% of the total, respectively. The cumulative contribution of these factors reached as high as 72.5%. In the analysis excluding host plants, Bio12 (24.6%, annual precipitation), Bio14 (19.1%, precipitation in the driest month), and Bio17 (15%, precipitation in the driest quarter) made the most critical contributions to the model. The cumulative contribution of these factors reached as high as 58.7% (Table 1).

The ranges of the five key environmental factors across different models, derived from the response curves (Figures S1 and S2), are listed in Table 2. Among these five factors, four were common to both models. The range of Bio4 (temperature seasonality) in the host plant model was 340.929 °C–810.569 °C, and that of Bio14 (precipitation in the driest month) in the plant-excluded model was 11.563 mm–217.800 mm. The mean temperature of the warmest quarter (Bio10) ranged from 16.306 °C to 30.871 °C in the plant model, and in the model with no plants, Bio14 ranged from 18.600 °C to 36.787 °C, higher than the other model. The range of annual precipitation (Bio12) was 790.275 mm to 4590.100 mm, larger than that in the no-plant model (844.773 to 3840.746 mm). Bio17 (precipitation in the driest quarter) ranged from 42.022 mm to 295.282 mm, lower than that in the no-plant model (44.702 mm to 375.208 mm), while Bio19 (precipitation in the coldest quarter) ranged from 44.620 mm to 390.910 mm in the plant model and from 47.244 mm to 389.230 mm in the no-plant model.

### 3.4. The Distribution of Highly Suitable Habitats for L. edodes

**Figure 3:**
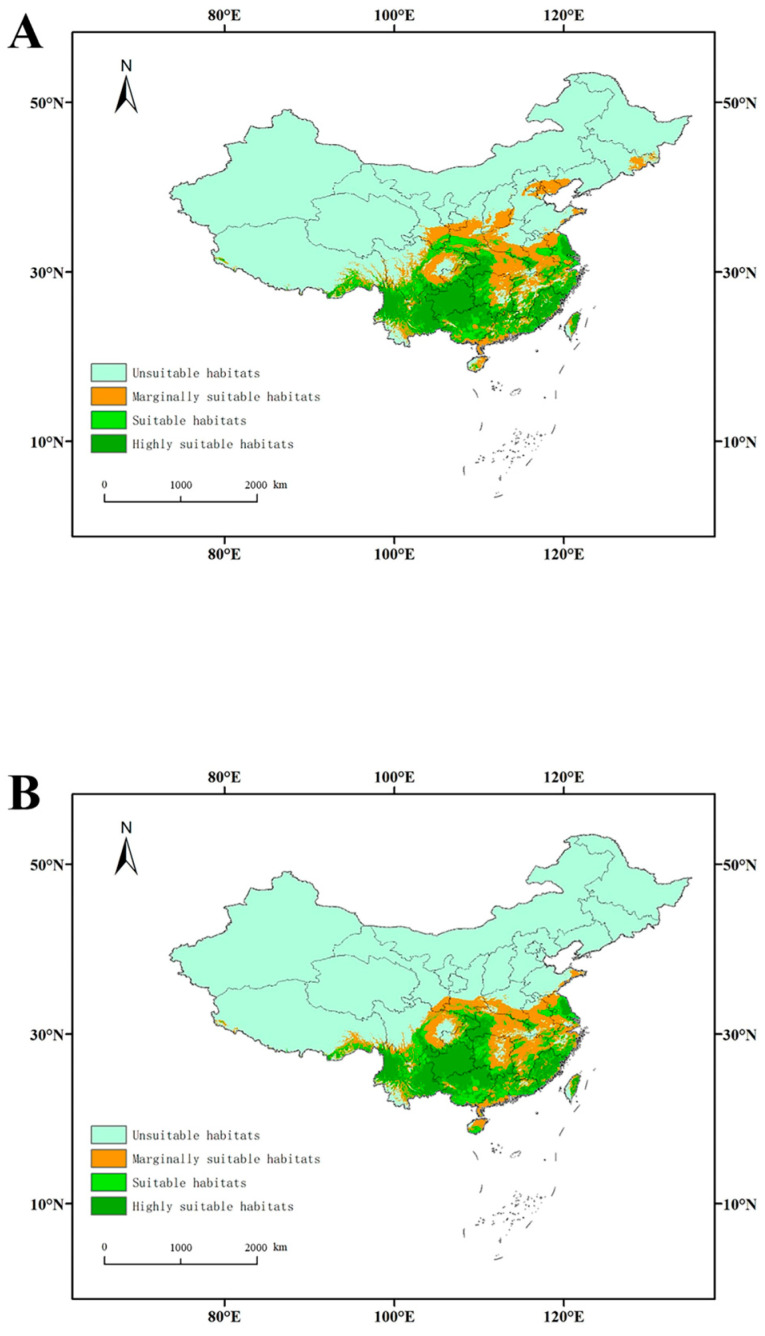
Habitat suitability for *L. edodes* in China: (**A**): six plant hosts; (**B**): no plants.

In the distribution model excluding host plant variables, the area of highly suitable habitats was 80.155 × 10^4^ km^2^, primarily distributed in southern and southwestern China (Table 3, Figure 3). The areas of suitable, marginally suitable, and unsuitable habitats were 51.315 × 10^4^, 93.368 × 10^4^, and 734.182 × 10^4^ km^2^, respectively (Table 3). However, when the factors related to plants were considered, all the areas of suitable, highly suitable, and marginally suitable habitats increased (Table 3). In the model featuring all plant taxa, the areas of suitable and highly suitable habitats were 55.386 × 10^4^ km^2^ and 88.493 × 10^4^ km^2^, respectively, representing 7.933% and 10.403% increases. These areas were primarily located in southern and southwestern China (Figure 3). After incorporating the vegetation factor, the overall distribution area shifted northward and westward.

### 3.5. Future Changes in the Geographical Distribution of L. edodes

**Figure 4:**
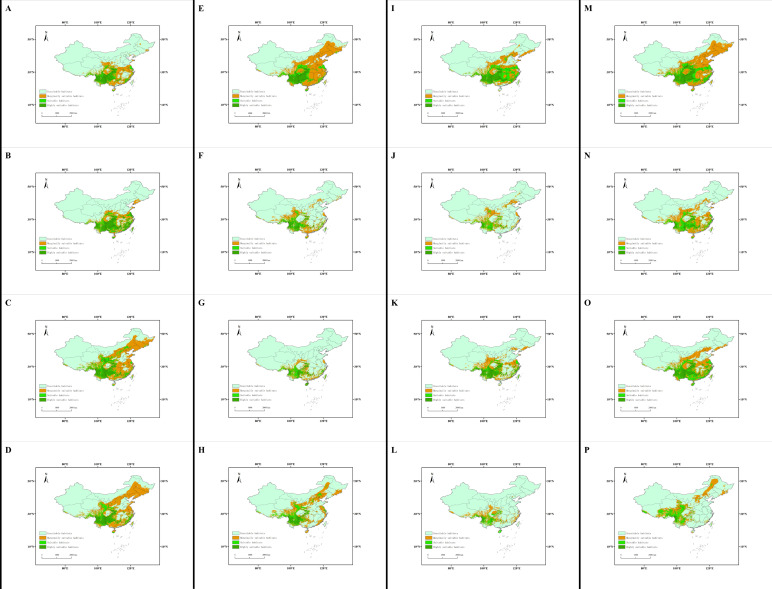
The distribution patterns of *L. edodes* under future scenarios: (**A**) 2030s in the SSP1-2.6 climate scenario; (**B**) 2050s in the SSP1-2.6 climate scenario; (**C**) 2070s in the SSP1-2.6 climate scenario; (**D**) 2090s in the SSP1-2.6 climate scenario; (**E**) 2030s in the SSP2-4.5 climate scenario; (**F**) 2050s in the SSP2-4.5 climate scenario; (**G**) 2070s in the SSP2-4.5 climate scenario; (**H**) 2090s in the SSP2-4.5 climate scenario; (**I**) 2030s in the SSP3-7.5 climate scenario; (**J**) 2050s in the SSP3-7.5 climate scenario; (**K**) 2070s in the SSP3-7.5 climate scenario; (**L**) 2090s in the SSP3-7.5 climate scenario; (**M**) 2030s in the SSP5-8.5 climate scenario; (**N**) 2050s in the SSP5 - 8.5 climate scenario; (**O**) 2070s in the SSP5-8.5 climate scenario; (**P**) 2090s in the SSP5-8.5 climate scenario.

Under four climate change scenarios (SSP 1-2.6, ssp2-4.5, ssp3-7.5 and SSP 5-8.5), the *L. edodes* distribution was predicted to decrease, with this decrease varying by area. Compared with the current potential distribution, the center of the suitable area shifted to the north and west. For the highly suitable habitat, the decrease in area ranged from 21.155% in the 2070s under the ssp1-2.6 scenario to 90.522% in the 2050s under the ssp3-7.5 scenario. The center of the distribution migrated from 239.533 km in the 2070s under the ssp2-4.5 scenario to 787.553 km in the 2090s under the ssp5-8.5 scenario (Figure 4).

## 4. Discussion

In this study, the geographical distribution of *L. edodes* was utilized to predict the suitable areas for this species in China, as well as their future changes, using the MaxEnt method. The results show that highly suitable areas are concentrated in the southwest of China, with the key environmental variables influencing the distribution of *L. edodes* including the host plants, temperature, and precipitation. In all 16 future scenarios, the area of highly suitable zones exhibited a decrease. Additionally, the center of high suitability was predicted to shift slightly to the northwest.

In this study, *L. edodes* was found to occur in 28 provinces in China. Compared with two other studies, some new locations were recorded, e.g., Beijing, Gansu, Hainan, Hebei, Shandong, and Xizang. However, there were still no records in Macao, Qinghai, Shanghai, Tianjin, or Hong Kong, as well as in some other places. In future work on wild *L. edodes*, emphasis should be placed on surveys in these regions, as they may also harbor an abundance of *L. edodes* that requires further study. These locations are important in the prediction of *L. edodes* distribution. The MaxEnt model is widely used in ecological research and plays an important role in predicting the potential distribution and environmental variables of animals and plants in their adaptive zones [24,25,26,27,28,29]. However, currently, there is limited research on the potential distribution adaptation zones and environmental variables affecting fungi based on this model [19,32,33,34,35,36,37,39]. This study represents the first attempt to predict the geographical distribution of the *L. edodes* population in China and uncover key environmental factors.

When predicting the potential distribution of *L. edodes*, it was found that the most suitable areas are primarily located in southern and southwestern regions of China, including Yunnan, Guizhou, Sichuan, Fujian, Guangdong, Zhejiang, Jiangsu, and others. This indicates that subtropical and tropical regions are optimal habitats for *L. edodes*, which is consistent with Hibbett’s research suggesting that *L. edodes* likely originated in tropical areas [5,17,40]. When predicting the potential distribution of *L. edodes*, accounting for the host plant factor, the contribution of host plants was consistently the highest, indicating that the distribution of *L. edodes* is greatly influenced by the distribution of host plants. This result is consistent with research on *T. matsutake*, *O. sinensis*, and *Sanghuangporus* mushrooms [34,36,37], in which it was found that *T. matsutake* and *O. sinensis* will not grow without suitable vegetation even if the climate conditions are favorable [28,34,35,36] and that *Sanghuangporus* exhibits host specificity, where the host plants restrict its distribution, playing a decisive role [37]. The host plants for *L. edodes*, such as the genera *Quercus, Castanopsis*, *Lithocarpus*, and *Castanea*, all belong to the family Fagaceae. In China, Fagaceae plants are widely distributed, except in Xinjiang. The richest diversity is observed in Yunnan, Guangxi, and Guangdong, gradually decreasing outwards from south to north, concentrated in the southwest region [43]. The distribution of these plants also determines the natural distribution of *L. edodes*.

In predictions of future geographical distributions, the distribution areas of *L. edodes* were largely reduced. Predictions of the future geographical distributions of the host genus *Quercus* are consistent with the current distribution patterns [44]; for *Quercus* species, with potential temperature rises in 2070, suitable distribution areas will expand to the north and the highly suitable areas will decrease by 6.44%. In addition to vegetation factors (i.e., host plants), which have the greatest impact, temperature- and precipitation-related bioclimatic variables are also important factors affecting the distribution of *L. edodes*: in our models, key temperature and precipitation variables (e.g., Bio10, Bio12, Bio17) contributed cumulatively up to 21% (with six host plants included) and 58.7% (with host plants excluded) to the model performance (Table 1). These factors also influence the geographical distribution of *T. matsutake*, *O. sinensis*, Boletes, and *Sanghuangporus* mushrooms [28,34,35,36,37]. Global warming has led to a decrease in the geographical distribution of *L. edodes*, with the most suitable areas shifting northward. These results are consistent with the prediction results for other mushrooms, except for *Sanghuangporus* and *P. umbellatus*, whose highly suitable habitat areas were observed to increase under four future scenarios [37,45]. Suitable areas for *P. umbellatus* are expected to increase in the northeast and southwest regions with a continuous rise in temperature, while they may also expand with a reduction in greenhouse gas emissions [45].

Outside China, the suitable habitats of *Tuber aestivum* (Wulfen) Spreng, *Tuber melanosporum* Vittad, and their ectomycorrhizal tree partners exhibit a northward shift. The suitable area has decreased significantly in Southern Europe but increased notably in Central and Northern Europe [46]. The current high-suitability habitats of *Ophiocordyceps sinensis* (Berk.) are mainly concentrated in India, China, Nepal, and Bhutan. Under all future SSP scenarios, its suitable habitats will decrease significantly and shift southwestward [47]. The suitable habitats of the North American invasive fungus *Aureoboletus projectellus* (Murrill) Halling in Europe form a continuous ring around the Baltic Sea. Currently, it has established populations in Poland and the Baltic countries, and is expected to further expand along the northern coast of the Baltic Sea in the future [48]. The suitable habitats of the North American false truffle *Rhizopogon salebrosus* A.H. Sm. in Europe are mainly concentrated in mountainous and peninsular areas, such as the western Iberian Peninsula and the British Isles. Currently, it has expanded from mountainous areas to the northern lowlands of Poland, and the suitable habitats in the lowlands may be underestimated [49]. The suitable habitats of *Hericium flagellum* (Scop.) Pers. in Europe overlap with the distribution of silver fir (which is concentrated in mountainous areas such as the Pyrenees and the Alps). Currently, it exhibits a discontinuous distribution, and is expected to shift to higher altitudes or northward alongside silver fir in the future [50]. The current suitable habitats of *Austropuccinia psidii* (G. Winter) Beenken in Australia are mainly concentrated in the eastern and southern coastal areas. In the future, its suitable habitats will expand slightly in the inland areas of New South Wales and Tasmania but shrink in northern Queensland and Western Australia [51]. The global suitable habitat diversity of fungi is characterized by concentration at high latitudes and lower diversity in the tropics. The habitats of most common fungi are climate-driven, and ectomycorrhizal fungi have narrower climatic niches than pathogenic fungi. Under climate change, their habitat distributions will adjust significantly with climate variables [52]. Climate is the core factor driving habitat changes in the above-mentioned fungi. Variables such as precipitation in the coldest quarter, annual mean temperature, and temperature seasonality dominate the migration of their habitats to higher latitudes or altitudes, or in specific directions.

*L. edodes* is a very popular edible fungus in Asia, especially in China, and it has received widespread attention due to its excellent nutritional and physiological activities. Therefore, it is necessary to model its geographical distribution in China. However, distribution records in northeastern, northern, and northwestern China are relatively scarce, which may limit our predictions. Given the global distribution of *L. edodes*, it is essential to predict its distribution on a global scale to clarify its distribution patterns, determine its relationships with host plants and living environments, and forecast changes in future suitable habitats as well as population dynamics. This could help us better utilize and protect *L. edodes* resources. In summary, there are three key factors: the availability of host plants, human harvesting activities, and maintenance of forest cover to contribute to adequate temperature and humidity. In this context, this study holds that the interaction of these factors with the continuous changes in wild mushroom resources has raised an important question: Given that *L. edodes* currently have the “Least Concern (LC)” status in the IUCN Red List, will a reassessment of this category be necessary in the future? Although this study has laid the foundation for understanding the distribution dynamics of *L. edodes*, explicitly incorporating the aforementioned factors and their potential impacts on the IUCN classification not only enriches the ecological and conservation perspectives of the research, but also points out the key directions for subsequent studies—ultimately providing more evidence-based strategies to support the sustainable utilization and conservation of *L. edodes* resources worldwide.

## Figures and Tables

**Table 1:** Environmental variables used to model the distribution of *L. edodes* and their contributions to the predictive model.

Variable	Description	Unit	Six Host Plants Percent Contribution (%)	Without Host Plant Percent Contribution (%)
hostplant	hostplant	-	51.5	0
Bio12	Annual precipitation	mm	4.8	24.6
Bio17	Precipitation in the driest quarter	mm	5.6	15
Bio14	Precipitation in the driest month	mm	3	19.1
Bio10	Mean temperature of the warmest quarter	°C	5.7	4.7
Bio19	Precipitation in the coldest quarter	mm	9.7	8.1
Bio7	Annual temperature range	°C	3.5	3.7
Bio4	Temperature seasonality	°C	3.8	3.9
Bio13	Precipitation in the wettest month	mm	1.1	2.9
Bio15	Precipitation seasonality	%	1.8	2.5
Bio3	Isothermality	%	1.5	2.7
Bio9	Mean temperature of the driest quarter	°C	2.1	2.4
Bio5	Max temperature of warmest month	°C	1.5	0.6
Bio8	Mean temperature of the wettest quarter	°C	0.7	1.8
Bio2	Mean diurnal range	°C	1.4	2.1
Bio16	Precipitation in the wettest quarter	mm	0.5	0.5
Bio6	Min temperature of the coldest month	°C	0.2	4.2
Bio11	Mean temperature of the coldest quarter	°C	0.8	0.7
Bio1	Annual mean temperature	°C	0.4	0.3
Bio18	Precipitation in the warmest quarter	mm	0.4	0.1
elev	Elevation	m	0	0

**Table 2:** Ranges of key environmental variables in highly suitable habitats under different host plant conditions.

Environmental Variable	All Host Plants	No Host Plants
Bio4	340.929–810.569 °C	-
Bio10	16.306–30.871 °C	18.600–36.787 °C
Bio12	790.275–4590.100 mm	844.773–3840.746 mm
Bio14	-	11.563–217.800 mm
Bio17	42.022–295.282 mm	44.702–375.208 mm
Bio19	44.620–390.910 mm	47.244–389.230 mm

**Table 3:** The predicted distribution area and migration distance of *L. edodes* under different suitability levels.

Model	Habitat Area (×10^4^ km^2^)/Variation Compared with the Current Area (%)				Mass Center Longitude	Mass Center Latitude	Mass Center Migration Distance (km)
	Unsuitable	Marginally Suitable	Suitable	Highly Suitable			
without host plants	734.182	93.368	51.315	80.155	110.438	27.559	-
with host plants	703.375/−4.196%	111.767/19.705%	55.386/7.933%	88.493/10.403%	109.936	28.482	106.224
ssp1-2.6 2030s	736.101/0.261%	130.356/39.615%	41.922/−18.305%	51.253/−36.057%	105.162	28.908	584.085
ssp1-2.6 2050s	816.304/11.185%	69.285/−25.794%	34.717/−32.345%	39.326/−50.937%	105.126	28.147	591.631
ssp1-2.6 2070s	561.745/−23.487%	256.917/175.165%	77.773/51.560%	63.198/−21.155%	104.739	29.677	654.677
ssp1-2.6 2090s	590.161/−19.617%	258.401/176.754%	57.925/12.882%	53.144/−33.698%	105.450	29.269	604.258
ssp2-4.5 2030s	536.941/−26.865%	299.597/220.876%	60.002/16.929%	63.092/−21.287%	105.246	29.024	634.026
ssp2-4.5 2050s	772.865/5.269%	114.245/22.359%	41.073/−19.959%	31.450/−60.764%	105.530	28.595	528.733
ssp2-4.5 2070s	830.630/13.137%	70.759/−24.216%	35.490/−30.840%	22.753/−71.613%	107.068	27.318	239.533
ssp2-4.5 2090s	700.193/−4.630%	170.736/82.863%	55.828/8.795%	32.875/−58.986%	104.140	29.916	730.958
ssp3-7.5 2030s	649.719/−11.504%	166.498/78.324%	82.109/60.011%	61.306/−23.516%	105.521	28.716	580.412
ssp3-7.5 2050s	815.912/11.132%	99.813/6.902%	36.311/−29.239%	7.597/−90.522%	105.867	29.228	542.587
ssp3-7.5 2070s	741.993/1.064%	121.247/29.858%	40.542/−20.994%	55.851/−30.322%	108.000	27.861	266.533
ssp3-7.5 2090s	843.566/14.899%	76.783/−17.763%	29.139/−43.215%	10.144/−87.344%	104.430	27.834	320.134
ssp5-8.5 2030s	518.653/−29.356%	282.844/202.933%	82.979/61.706%	75.156/−6.236%	105.647	28.932	578.737
ssp5-8.5 2050s	696.969/−5.069%	164.559/76.247%	72.667/41.609%	25.438/−68.265%	107.225	28.550	248.502
ssp5-8.5 2070s	655.629/−10.700%	160.722/72.138%	78.160/52.314%	65.122/−18.755%	106.410	28.435	430.323
ssp5-8.5 2090s	745.882/1.594%	135.865/45.515%	66.483/29.558%	11.403/−85.774%	103.404	29.655	787.553

## Data Availability

The original contributions presented in this study are included in the article. Further inquiries can be directed to the corresponding authors.

## Supplementary Materials

The following supporting information can be downloaded at https://www.mdpi.com/article/10.3390/jof11100730/s1: Figures S1 and S2: Response curves of existence probability of *L. edodes* distribution model with host plants and excluding host plants; Table S1: Geographic distribution points of *Lentinula edodes* in China; Table S2: Geographical distributions of *Lentinula edodes* species sampled used in this study.

## References

[1] Pegler D.N. (1983). The genus *Lentinula* (Tricholomataceae tribe Collybieae). Sydowia.

[2] Mata J.L., Petersen R.H., Hughes K.W. (2001). The genus *Lentinula* in the Americas. Mycologia.

[3] Liu Q., Niu S., Hu S., Cui X., Shi Z., Wu J., Zhang Y., Kong W. (2023). Lignocellulose degradation pattern and structural change of the sawdust substrate and enzyme secretion by *Lentinula edodes* during its production. Wood Sci. Technol..

[4] Xu S., Wang F., Fu Y., Li D., Sun X., Li C., Song B., Li Y. (2020). Effects of mixed agro-residues (corn crop waste) on lignin-degrading enzyme activities, growth, and quality of *Lentinula edodes*. RSC Adv..

[5] Sierra-Patev S., Min B., Naranjo-Ortiz M., Looney B., Konkel Z., Slot J.C., Sakamoto Y., Steenwyk J.L., Rokas A., Carro J. (2023). A global phylogenomic analysis of the shiitake genus *Lentinula*. Proc. Natl. Acad. Sci. USA.

[6] Song X., Shang X., Zhang M., Yu H., Zhang D., Tan Q., Song C. (2025). Cultivation methods and biology of *Lentinula edodes*. Appl. Microbiol. Biotechnol..

[7] Bugajewski M., Angerhoefer N., Pączek L., Kaleta B. (2025). *Lentinula edodes* as a source of bioactive compounds with therapeutical potential in intestinal inflammation and colorectal cancer. Int. J. Mol. Sci..

[8] Roszczyk A., Turło J., Zagożdżon R., Kaleta B. (2022). Immunomodulatory properties of polysaccharides from *Lentinula edodes*. Int. J. Mol. Sci..

[9] Singh A., Saini R.K., Kumar A., Chawla P., Kaushik R. (2025). Mushrooms as nutritional powerhouses: A review of their bioactive compounds, health benefits, and value-added products. Foods.

[10] Chang S.T., Miles P.G. (1987). Historical record of the early cultivation of *Lentinus* in China. Mushroom J. Trop..

[11] Yang R.H., Wu Y.Y., Song C.Y., Guo T., Li C.H., Tang L.H., Bao D.P. (2018). Reanalysis of geographical distribution of wild *Lentinula edodes* in China based on the information from public databases. Mycosystema.

[12] Yang R.H., Wu Y.Y., Tang L.H., Li C.H., Shang J.J., Li Y., Song Y., Huang W.H., Tao X.S., Tan Q. (2019). The reliability of DNA Sequences in public databases belonging to the most economically important shiitake culinary-medicinal mushroom *Lentinus edodes* (Agaricomycetes) in Asia. Int. J. Med. Mushrooms.

[13] Liu J., Wang Z.R., Li C., Bian Y.B., Xiao Y. (2015). Evaluating genetic diversity and constructing core collections of Chinese *Lentinula edodes* cultivars using ISSR and SRAP markers. J. Basic. Microbiol..

[14] Liu J.Y., Ying Z.H., Liu F., Liu X.R., Xie B.G. (2012). Evaluation of the use of SCAR markers for screening genetic diversity of *Lentinula edodes* strains. Curr. Microbiol..

[15] Song X., Zhao Y., Song C., Chen M., Huang J., Bao D., Tan Q., Yang R. (2019). Mitogenome types of two *Lentinula edodes* sensu lato populations in China. Sci. Rep..

[16] Yu H., Zhang L., Shang X., Peng B., Li Y., Xiao S., Tan Q., Fu Y. (2022). Chromosomal genome and population genetic analyses to reveal genetic architecture, breeding history and genes related to cadmium accumulation in *Lentinula edodes*. BMC Genom..

[17] Zhang J., Shen N., Li C., Xiang X., Liu G., Gui Y., Patev S., Hibbett D.S., Barry K., Andreopoulos W. (2022). Population genomics provides insights into the genetic basis of adaptive evolution in the mushroom-forming fungus *Lentinula edodes*. J. Adv. Res..

[18] Grimm N.B., Chapin Iii F.S., Bierwagen B., Gonzalez P., Groffman P.M., Luo Y., Melton F., Nadelhoffer K., Pairis A., Raymond P.A. (2013). The impacts of climate change on ecosystem structure and function. Front. Ecol. Environ..

[19] Coelho M.T.P., Barreto E., Rangel T.F., Diniz-Filho J.A.F., Wüest R.O., Bach W., Skeels A., McFadden I.R., Roberts D.W., Pellissier L. (2023). The geography of climate and the global patterns of species diversity. Nature.

[20] Zu K., Wang Z., Zhu X., Lenoir J., Shrestha N., Lyu T., Luo A., Li Y., Ji C., Peng S. (2021). Upward shift and elevational range contractions of subtropical mountain plants in response to climate change. Sci. Total Environ..

[21] Lenoir J., Svenning J.C. (2013). Latitudinal and elevational range shifts under contemporary climate change. Encyclopedia of Biodiversity.

[22] Brodie J.F., Freeman B.G., Mannion P.D., Hargreaves A.L. (2025). Shifting, expanding, or contracting? Range movement consequences for biodiversity. Trends Ecol. Evol..

[23] Yesuf G.U., Brown K.A., Walford N.S., Rakotoarisoa S.E., Rufino M.C. (2021). Predicting range shifts for critically endangered plants: Is habitat connectivity irrelevant or necessary?. Biol. Conserv..

[24] Liu B., Gao X., Ma J., Jiao Z., Xiao J., Hayat M.A., Wang H. (2019). Modeling the present and future distribution of arbovirus vectors *Aedes aegypti* and *Aedes albopictus* under climate change scenarios in Mainland China. Sci. Total Environ..

[25] Liu T., Wang J., Hu X., Feng J. (2020). Land-use change drives present and future distributions of fall armyworm, *Spodoptera frugiperda* (J.E. Smith) (Lepidoptera: Noctuidae). Sci. Total Environ..

[26] Qiu L., Jacquemyn H., Burgess K.S., Zhang L.G., Zhou Y.D., Yang B.Y., Tan S.L. (2023). Contrasting range changes of terrestrial orchids under future climate change in China. Sci. Total Environ..

[27] Wang Y., Ma X., Lu Y., Hu X., Lou L., Tong Z., Zhang J. (2022). Assessing the current genetic structure of 21 remnant populations and predicting the impacts of climate change on the geographic distribution of *Phoebe sheareri* in southern China. Sci. Total Environ..

[28] Wei Y., Zhang L., Wang J., Wang W., Niyati N., Guo Y., Wang X. (2021). Chinese caterpillar fungus (*Ophiocordyceps sinensis*) in China: Current distribution, trading, and futures under climate change and overexploitation. Sci. Total Environ..

[29] Xu L., Fan Y., Zheng J., Guan J., Lin J., Wu J., Liu L., Wu R., Liu Y. (2024). Impacts of climate change and human activity on the potential distribution of *Aconitum leucostomum* in China. Sci. Total Environ..

[30] Zhang K., Yao L., Meng J., Tao J. (2018). Maxent modeling for predicting the potential geographical distribution of two *peony* species under climate change. Sci. Total Environ..

[31] Ke C., Gong L.X., Geng Y., Wang Z.Q., Zhang W.J., Feng J., Jiang T.L. (2025). Patterns and correlates of potential range shifts of bat species in China in the context of climate change. Conserv. Biol..

[32] Chen J., He D. (2024). Potential geographical distribution of *Cordyceps cicadae* and its two hosts in China under climate change. Front. Microbiol..

[33] Alkhalifah D.H.M., Damra E., Melhem M.B., Hozzein W.N. (2023). Fungus under a changing climate: Modeling the current and future global distribution of *Fusarium oxysporum* using geographical information system data. Microorganisms.

[34] Guo Y., Li X., Zhao Z., Wei H., Gao B., Gu W. (2017). Prediction of the potential geographic distribution of the ectomycorrhizal mushroom *Tricholoma matsutake* under multiple climate change scenarios. Sci. Rep..

[35] Pradhan P., Deshmukh S.K., Sridhar K.R. (2024). Biogeography and impacts of climate change on the distribution of *Ophiocordyceps sinensis*. Cordyceps and Allied Species.

[36] Yan Y., Li Y., Wang W.-J., He J.-S., Yang R.-H., Wu H.-J., Wang X.-L., Jiao L., Tang Z., Yao Y.-J. (2017). Range shifts in response to climate change of *Ophiocordyceps sinensis*, a fungus endemic to the Tibetan Plateau. Biol. Conserv..

[37] Chen J.H., Shen S., Zhou L.W. (2022). Modeling current geographic distribution and future range shifts of *Sanghuangporus* under multiple climate change scenarios in China. Front. Microbiol..

[38] Cohen S.D. (2023). Estimating the climate niche of *sclerotinia sclerotiorum* using maximum entropy modeling. J. Fungi.

[39] Schunko C., Li X., Klappoth B., Lesi F., Porcher V., Porcuna-Ferrer A., Reyes-García V. (2022). Local communities’ perceptions of wild edible plant and mushroom change: A systematic review. Glob. Food Secur..

[40] Menolli Jr N., Sánchez-Ramírez S., Sánchez-García M., Wang C., Patev S., Ishikawa N.K., Mata J.L., Lenz A.R., Vargas-Isla R., Liderman L. (2022). Global phylogeny of the Shiitake mushroom and related *Lentinula* species uncovers novel diversity and suggests an origin in the Neotropics. Mol. Phylogenet. Evol..

[41] Phillips S.J., Anderson R.P., Dudík M., Schapire R.E., Blair M.E. (2017). Opening the black box: An open-source release of Maxent. Ecography.

[42] Halvorsen R., Mazzoni S., Dirksen J.W., Næsset E., Gobakken T., Ohlson M. (2016). How important are choice of model selection method and spatial autocorrelation of presence data for distribution modelling by MaxEnt?. Ecol. Model..

[43] Zhu J., Ji C., Zhang H., Ran Q., Tao S., Wang Z., Xu X., Cai Q., Fang J. (2025). Possible refugia for Fagaceae species in China under climate change. J. Plant Ecol..

[44] Wang X., Duan Y., Jin L., Wang C., Peng M., Li Y., Wang X., Ma Y. (2023). Prediction of historical, present and future distribution of *Quercus sect*. Heterobalanus based on the optimized MaxEnt model in China. Acta Ecol. Sin..

[45] Guo Y., Li X., Zhao Z., Nawaz Z. (2019). Predicting the impacts of climate change, soils and vegetation types on the geographic distribution of *Polyporus umbellatus* in China. Sci. Total Environ..

[46] Wilgan R., Dyderski M.K., Pietras M., Walas Ł., Kolanowska M., Leski T. (2025). Northward shifting in the distribution of optimal niches for *Tuber aestivum*, *Tuber melanosporum*, and their ectomycorrhizal tree partners in Europe. Acta Oecologica.

[47] Salam N., Sidhu H.K., Shaban S., Reshi Z.A., Shah M.A. (2025). Climate change scenarios predict reduction in suitable habitats and range shifts for *Ophiocordyceps sinensis* (Berk.) in Hindu Kush Himalaya. J. Asia-Pac. Biodivers..

[48] Banasiak Ł., Pietras M., Wrzosek M., Okrasińska A., Gorczak M., Kolanowska M., Pawłowska J. (2019). *Aureoboletus projectellus* (Fungi, Boletales)—Occurrence data, environmental layers and habitat suitability models for North America and Europe. Data Brief.

[49] Pietras M., Kolanowska M. (2019). Predicted potential occurrence of the North American false truffle *Rhizopogon salebrosus* in Europe. Fungal Ecol..

[50] Kujawska M.B., Rudawska M., Stasińska M., Pietras M., Leski T. (2021). Distribution and ecological traits of a rare and threatened fungus *Hericium flagellum* in Poland with the prediction of its potential occurrence in Europe. Fungal Ecol..

[51] Berthon K., Esperon-Rodriguez M., Beaumont L.J., Carnegie A.J., Leishman M.R. (2018). Assessment and prioritisation of plant species at risk from myrtle rust (*Austropuccinia psidii*) under current and future climates in Australia Biological Conservation. Biol. Conserv..

[52] Větrovský T., Kohout P., Kopecký M., Macháč A., Man M., Bahmann B.D., Brabcová V., Choi J., Meszárošová L., Human Z.R. (2019). A meta-analysis of global fungal distribution reveals climate-driven patterns. Nat. Commun..

