# Supplementary material for: A Model of the Current Geographic Distribution and Predictions of Future Range Shifts of Lentinula edodes in China Under Multiple Climate Change Scenarios
Source: J Fungi (Basel). 2025 Oct 10;11(10):730. doi: 10.3390/jof11100730 (PMC12565594; doi:10.3390/jof11100730)
Supplement: Supplementary file 1 [file jof-11-00730-s001.zip › Table S1. Geographic distribution points of Lentinula edodes in China.pdf]

Table S1 Geographic distribution points of *Lentinula edodes* in China

Region	Province	Prefecture-level city	County-level city	Location
North China Region	Beijing	Beijing	-	Huairou Distrct
	Hebei	Chengde	Xinglong County	Wuling Mountain National Nature Reserve
		Tangshan	-	-
		Qinhuangdao	-	-
Northeast China Region	Heilongjiang	Daqing	-	-
		Lesser Khingan shans	-	-
	Jilin	Jilin	Huadian City	Dayang Cha
		Yanbian Chaoxianzu Autonomous Prefecture	Antu County	Erdaobaihe Town
		Changbai shans	-	-
	Liaoning	Benxi	-	-
		Benxi	Huanren Manchu Autonomous County	-
		Shenyang	-	-
		Dalian	-	San Yue Da Bio-Technology Co., Ltd
		Fushun	Xinbin manchu Autonomous County	Gang Mountain National Forest Park
East China Region	Zhejiang	Lishui	-	-
		Lishui	Jingning She Autonomous County	-
		Lishui	-	-

	Lishui	Yunhe County	-
	Lishui	Qingyuan County	-
	Lishui	Longquan City	-
	Lishui	Suichang County	Hu Mountain Forest Park
	Lishui	Longquan City	Fengyang Mountain National Nature Reserve
	Zhejiang	-	Yuhang District
	Wenzhou	-	-
	Wenzhou	Taishun County	-
	Quzhou	Kaihua County	Gutian Mountain guo National Nature Reserve
	Hangzhou	-	Linan District Tianmu Mountain Scenic Spots
Anhui	Anqing	Yuexi County	Laibang Town
	Anqing	Yuexi County	Shiguan Village
	Huangshan	-	Huang Mountain
	Huangshan	Xiuning County	-
	Huangshan	Yi County	-
	lu'an	Jinzhai County	-
	lu'an	Jinzhai County	Banzhuyuan Town
	lu'an	Shucheng County	-
	lu'an	HuoMountain County	-
	Suzhou	Lingbi County	-
	Xuancheng	-	Xuanzhou District
	Hefei	-	-
	shans in southern Anhui	-	-

	Ta-pieh shans	-	-
	Jianghuai hilly region	-	-
Jiangxi	Ji'an	JinggangMountain City	-
	Ji'an	JinggangMountain City	Ciping Town
	Ji'an	JinggangMountain City	Lanhuaping
	Ji'an	JinggangMountain City	Wuzhifeng
	Ji'an	JinggangMountain City	Jingzhu Mountain
	Ji'an	JinggangMountain City	Xiaojinglongtan
	Shangrao	YuMountain County	-
	Shangrao	Dexing City	Damao Mountain
	Shangrao	Dexing City	Damao Mountain
	Shangrao	Poyang County	-
	Shangrao	YanMountain County	-
	Ganzhou	-	-
	Ganzhou	Xinfeng County	-
	Ganzhou	Mountaingyou County	-
	Ganzhou	Shicheng County	Ganjiangyuan Nature Reserve
	Yingtan	-	-
	Yingtan	Guixi City	-
	Pingxiang	Luxi County	Wugong Mountain
	Pingxiang	-	Anyuan District
	Fuzhou	-	-
	Fuzhou	Lichuan County	Rock Springs National Forest Park
	Jiujiang	Xiushui County	-
	Yichun	-	-

		-	-	-
	Shandong	Taian	-	Culai Mountain National Forest Park
	Fujian	Fuzhou	-	-
		Fuzhou	Minqing County	Huangchu Forest National Nature Reserve
		Fuzhou	Minhou County	Yang Mountain Village
		Nanping	-	-
		Nanping	-	Huangkeng Town
		Nanping	WuyiMountain City	Mount Wuyi Dazhugang
		Nanping	-	Jianyang District
		Nanping	Shunchang County	-
		Longyan	Wuping County	-
		Longyan	-	Longyan District
		Quanzhou	-	Counties in the Jinjiang Region
		Ningde	Gutian County	-
		Ningde	Gutian County	Qilin Mountain Park
		Sanming	-	Sanming District
		Sanming	Jianning County	-
		Sanming	JiangLe County	-
		Sanming	Mingxi County	-
		Sanming	Qingliu County	-
		Sanming	-	Shaxian Distrct
	Taiwan	Nantou	Nantou County	-
		-	-	-
Central China	Henan	Luoyang	-	-

Reigon				
		Luoyang	-	Yanshi Distrct
		Luoyang	Xinan County	-
		Luoyang	Luanchuan County	Jiaohe Town
		Luoyang	LuanMountain County	-
		Xinyang	-	Kikung Mountain National Nature Reserve
		Nanyang	Xixia Country	-
		Pingdingshan	-	-
		Sanmenxia	-	-
		Ta-pieh shans	-	-
	Hubei	Jingmen	-	Lixi Town
		Jingmen	JingMountain City	Sanyang Town
		Jingmen	JingMountain City	Sanyang Town
		Yichang	Wufeng Tujia Autonomous County	-
		Yichang	XingMountain County	-
		Yichang	Yuanan County	-
		Yichang	-	-
		Enshi Tujia and Miao Autonomous Prefecture	Xianfeng County	-
		Enshi Tujia and Miao Autonomous Prefecture	Enshi City	-
		Enshi Tujia and Miao Autonomous Prefecture	Hefeng County	-
		Xiangyang	Baokang County	-

		Shiyan	Zhuxi County	-
		Suizhou	-	-
		Shennongjia Forestry District	-	-
		Shennongjia Forestry District	-	-
	Hunan	Zhangjiajie	-	-
		Shaoyang	-	-
		Chenzhou	-	Mang Mountain National Forest Park
		Chenzhou	-	-
		Yongzhou	Jianghua Yao Autonomous County	Mashi Town Tiandeng
		Shaoyang	Wugang City	Yun Mountain National Forest Park
		Xiangxi Tujia and Miao Autonomous Prefecture	-	Fenghuang Mountain National Forest Park
South China Region	Guangdong	Tsingyün	-	-
		Tsingyün	Yingde City	Shimentai Nature Reserves
		Tsingyün	-	Yinghong Town Shuitou Village Wangwei Zu Aobei
		Tsingyün	YangMountain County	-
		Shaoguan	-	Qujiang District Xiaokeng Forest
		Shaoguan	Shixing County	Zhangdongshui
		Shaoguan	-	Dungang Town

	Shaoguan	-	Shirenzhang Town
	Shaoguan	-	Chebaling National Nature Reserve
	Shaoguan	-	Chebaling National Nature Reserve Xianren Cave
	Shaoguan	-	Chebaling National Nature Reserve Sanjiaotang
	Meizhou	Dabu County	Fengxi
	Chaozhou	-	Fenghuang Town Fenghuang Tianchi
Guangxi	Baise	-	-
	Baise	Napo County	-
	Baise	-	Diding Village
	Guilin	-	-
	Guilin	Lipu City	-
	Guilin	-	Lingui Huaping National Nature Reserve
	Guilin	-	Longsheng Huaping National Nature Reserve
	Hechi	-	-
	Hechi	Luocheng Mulam Autonomous County	luo cheng xian
	Hechi	Tian'e County	tian e xian
	Hechi	Nandan County	nan dan xian
	Hechi	Donglan County	dong lan xian

		Nanning	-	Daming Mountain National Nature Reserve
		Laibin	-	-
		Laibin	Jinxiu Yao Autonomous County	Dayao Mountain National Nature Reserve
		Fangchenggang	-	-
		Fangchenggang	-	Shiwanda Mountain National Nature Reserve
		Fangchenggang	Mountaingsi County	-
		Dayao shan	-	-
		-	-	-
	Hainan	Wuzhishan	-	-
Southwest China Region	Chongqing	Chongqing	-	Nanchuan District
		Chongqing	-	Wanchuan District
		Chongqing	Pengshui Miao&Tujia Autonomous County	-
		Chongqing	-	Jinfo Mountain National Nature Reserve
		Chongqing	-	Simian Mountain
	Sichuan	Mianyang	An District	-
		Mianyang	Beichuan Qiang Autonomous County	-
		Mianyang	Santai County	-
		Mianyang	Pingwu County	-
		Mianyang	-	-

Dazhou	-	Dachuan District
Dazhou	Wanyuan City	-
Bazhong	Tongjiang County	-
Bazhong	Nanjiang County	-
Ya'an	Jiangyuan County	-
Ya'an	Shimian County	-
Ya'an	-	-
Panzhihua	Yanbian County	-
Panzhihua	Miyi County	Malong Yi Autonomous Village
Panzhihua	Miyi County	-
Panzhihua	-	-
Guangyuan	Qingchuan County	-
Guangyuan	-	-
Liangshan Yi Autonomous Prefecture	Huidong County	-
Liangshan Yi Autonomous Prefecture	Huili City	-
Liangshan Yi Autonomous Prefecture	Dechang County	-
Liangshan Yi Autonomous Prefecture	Xichang City	Luoji Mountain
Liangshan Yi Autonomous Prefecture	Xichang City	Anha Town
Liangshan Yi Autonomous Prefecture	Xide County	-
Liangshan Yi	Ganluo County	-

	Autonomous Prefecture		
	Liangshan Yi Autonomous Prefecture	Mianning County	-
	Liangshan Yi Autonomous Prefecture	Muli Tibetan Autonomous County	-
	Liangshan Yi Autonomous Prefecture	Ningnan County	-
	Liangshan Yi Autonomous Prefecture	Meigu County	-
	Liangshan Yi Autonomous Prefecture	Puge County	-
	Liangshan Yi Autonomous Prefecture	Yanyuan County	-
	Liangshan Yi Autonomous Prefecture	Shaojue County	-
	Liangshan Yi Autonomous Prefecture	-	-
	Ganzi Tibetan Autonomous Prefecture	Luding County	Detuo Town
	Ganzi Tibetan Autonomous Prefecture	Jiulong County	-
	-	-	-
Guizhou	Zunyi	-	-
	Zunyi	Daozhen Gelaozu Miaozu Autonomous County	Dasha River Nature Reserve
	Zunyi	Tongxin County	-

Zunyi	Yuqing County	-
Zunyi	-	Kuankuoshui Forest National Nature Reserve
Zunyi	-	-
Zunyi	Zhengan County	-
Tongren	Jinagkou County	-
Tongren	Shiqian County	-
Tongren	-	Fanjing Mountain
Tongren	-	-
Bijie	Jinsha County	-
Bijie	-	Bailidujuan Nature Reserve
Bijie	Nayong County	-
Bijie	-	-
Guiyang	-	Baiyun Cattle Farm
Qianxinan Buyi and Miao Autonomous Prefecture	Ceheng County	-
Qianxinan Buyi and Miao Autonomous Prefecture	Wangmo County	-
Qiandongnan Miao and Dong Autonomous Prefecture	Liping County	-
Qiandongnan Miao and Dong Autonomous Prefecture	Leishan County	-
Qiandongnan Miao and Dong Autonomous	Jianhe County	-

Prefecture		
Qiandongnan Miao and Dong Autonomous Prefecture	Rongjiang County	-
Qiandongnan Miao and Dong Autonomous Prefecture	Jinping County	-
Qiandongnan Miao and Dong Autonomous Prefecture	Jianhe County	-
Qiandongnan Miao and Dong Autonomous Prefecture	Tianzhu County	-
Qiannan Buyi and Miao Autonomous Prefecture	Libo County	Maolan Forest National Nature Reserve
Qiannan Buyi and Miao Autonomous Prefecture	Luodian County	-
Qiannan Buyi and Miao Autonomous Prefecture	Huishui County	-
Qiannan Buyi and Miao Autonomous Prefecture	Libo County	-
Qiannan Buyi and Miao Autonomous Prefecture	Wengan County	-
Qiannan Buyi and Miao Autonomous Prefecture	Sandu Shui Autonomous County	-
Qiannan Buyi and Miao	Duyun City	Lvyin Lake Street

	Autonomous Prefecture		
	-	-	-
	-	-	-
	-	-	-
	-	-	-
Yunnan	Kunming	Xundian Hui and Yi Autonomous County	-
	Kunming	Yiliang County	-
	Kunming	Songming County	-
	Kunming	Luquan Yi and Miao Autonomous County	-
	Kunming	-	Qinglong Gorge
	Kunming	-	Yeya Lake
	Kunming	-	-
	Lijiang	Huaping County	-
	Lijiang	Yulong Naxi Autonomous County	Judian Town
	Lijiang	Ninglang Yi Autonomous County	-
	Lijiang	-	Yulong Mountain Baisha River
	Lijiang	Maguan County	Laojun Mountain
	Lijiang	Maguan County	Doulong Town Donggualin Village After the Mountain
	Lijiang	-	High Mountain Botanical Garden
	Lijiang	-	-

Qujing	Malong County	-
Qujing	-	-
Qujing	Shizong County	-
Qujing	Xuanwei City	-
Baoshan	Tengchong County	-
Baoshan	Changning County	-
Baoshan	Shidian County	-
Baoshan	Longling County	Tuantianxiangheng Mountain Village
Baoshan	Longling County	Xiaohei Mountain - Daxue Mountain Nature Reserve
Baoshan	Longling County	Gaoligong Mountain Forest Park
Baoshan	-	Luobo Yakou
Puer	-	Simao District
Puer	Mojiang Hani Autonomous County	-
Nantong	Zhaotong city	-
Meizhou	Wuhua County	Weixi Village
Chuxiong Yi Autonomous Prefecture	Wuding County	-
Chuxiong Yi Autonomous Prefecture	Chuxiong City	-
Chuxiong Yi Autonomous Prefecture	Lufeng County	-
Honghe Hani and Yi	Hekou Yao Autonomous	-

Autonomous Prefecture	County	
Dehong Dai and Jingpo Autonomous Prefecture	Longchuan County	-
Dehong Dai and Jingpo Autonomous Prefecture	Yingjiang County	-
Wenshan Zhuang and Miao Autonomous Prefecture	Guangnan County	-
Wenshan Zhuang and Miao Autonomous Prefecture	Qiubei County	-
Wenshan Zhuang and Miao Autonomous Prefecture	Wenshan County	-
Wenshan Zhuang and Miao Autonomous Prefecture	Maguan County	-
Dali Bai Autonomous Prefecture	Yongping County	-
Dali Bai Autonomous Prefecture	Binchuan County	-
Dali Bai Autonomous Prefecture	Yangbi Yi Autonomous County	-
Dali Bai Autonomous Prefecture	Dali City	-

	Dali Bai Autonomous Prefecture	-	Diancang Mountain
	Dali Bai Autonomous Prefecture	Weishan Yizu Huizu Autonomous County	-
	Dali Bai Autonomous Prefecture	Dali City	Xiaguan Town
	Diqing Tibetan Autonomous Prefecture	Deqin County	-
	Diqing Tibetan Autonomous Prefecture	-	-
	Diqing Tibetan Autonomous Prefecture	Deqin County	Benzilan
	Diqing Tibetan Autonomous Prefecture	Weixi Lisu Autonomous County	-
	Diqing Tibetan Autonomous Prefecture	Zhongdian County	-
	Nujiang Lisu Autonomous Prefecture	Bijiang County	-
	Ailaoshan National Nature Reserve	-	-
Tibet Autonomous Region	Nyingchi	Linzhi County	-
	Nyingchi	Motuo County	-
	Nyingchi	Chayu County	-
	Nyingchi	Chayu County	Xiachayu Town

Northwest China Region	Shaanxi	Ankang	-	-
		Ankang	Zhenping County	-
		Hanzhong	Lvyang County	-
		Hanzhong	Liuba County	-
		Hanzhong	-	Nanzheng District
		Hanzhong	-	-
		Qinling shans	-	-
		-	-	-
	Gansu	Longnan	-	-
		Longnan	Kang County	-
		Longnan	-	Wudu District
		Longnan	Wen County	-
		Longnan	-	Yangba
		Gannan Tibetan Autonomous Prefecture	-	Diebu
		Gannan Tibetan Autonomous Prefecture	Zhouqu County	-
		Taohe Forest Region	-	-
		Bailong river	-	-
