# Supplementary material for: A Model of the Current Geographic Distribution and Predictions of Future Range Shifts of Lentinula edodes in China Under Multiple Climate Change Scenarios
Source: J Fungi (Basel). 2025 Oct 10;11(10):730. doi: 10.3390/jof11100730 (PMC12565594; doi:10.3390/jof11100730)
Supplement: Supplementary file 1 [file jof-11-00730-s001.zip › Table S2. Geographical distributions of Lentinula. edodes species sampled used in this study.pdf]

Table S2. Geographical distributions of *Lentinula. edodes* species sampled used in this study

Species	Longitude (◦E)	Latitude (◦N)
Lentinula. edodes	99.09	26.1
Lentinula. edodes	99.29	27.56
Lentinula. edodes	99.53	25.19
Lentinula. edodes	99.61	25.73
Lentinula. edodes	99.88	25.68
Lentinula. edodes	100.04	25.71
Lentinula. edodes	100.32	25.49
Lentinula. edodes	102.36	27.67
Lentinula. edodes	102.38	25.19
Lentinula. edodes	102.42	25.04
Lentinula. edodes	102.53	34.59
Lentinula. edodes	104.16	24.64
Lentinula. edodes	104.41	32.58
Lentinula. edodes	104.95	24.62
Lentinula. edodes	105.36	33.2
Lentinula. edodes	106.23	23.12
Lentinula. edodes	106.48	23.24
Lentinula. edodes	107.01	33.04
Lentinula. edodes	107.07	25.13
Lentinula. edodes	107.22	24.32
Lentinula. edodes	107.33	25.27
Lentinula. edodes	107.37	26.37
Lentinula. edodes	107.58	22.09
Lentinula. edodes	107.95	21.87
Lentinula. edodes	107.95	25.93
Lentinula. edodes	108.58	23.02
Lentinula. edodes	108.63	26.11
Lentinula. edodes	108.77	27.84
Lentinula. edodes	109.22	25.05
Lentinula. edodes	109.26	27.61
Lentinula. edodes	109.31	31.53
Lentinula. edodes	109.31	18.46
Lentinula. edodes	109.92	25.78
Lentinula. edodes	110.4	31.45
Lentinula. edodes	110.52	30.57
Lentinula. edodes	110.54	25
Lentinula. edodes	110.55	24.32
Lentinula. edodes	110.63	25.98
Lentinula. edodes	110.97	25.4
Lentinula. edodes	111.29	33.18
Lentinula. edodes	111.51	36.27

Lentinula. edodes	112.07	24.85
Lentinula. edodes	112.5	24.59
Lentinula. edodes	116.09	27.23
Lentinula. edodes	116.37	40.19
Lentinula. edodes	116.49	25.13
Lentinula. edodes	116.65	23.95
Lentinula. edodes	119.44	30.34
Lentinula. edodes	119.75	30.54
Lentinula. edodes	120.18	30.26
Lentinula. edodes	123.54	41.51
Lentinula. edodes	125.01	46.36
Lentinula. edodes	128.37	42.25
Lentinula. edodes	119.21	27.75
