# Supplementary material for: A Model of the Current Geographic Distribution and Predictions of Future Range Shifts of Lentinula edodes in China Under Multiple Climate Change Scenarios
Source: J Fungi (Basel). 2025 Oct 10;11(10):730. doi: 10.3390/jof11100730 (PMC12565594; doi:10.3390/jof11100730)
Supplement: Supplementary file 1 [file jof-11-00730-s001.zip › Figure S1-S2. Response curves of existence probability of L. edodes distribution model with host plants and excluding host plants.pdf]

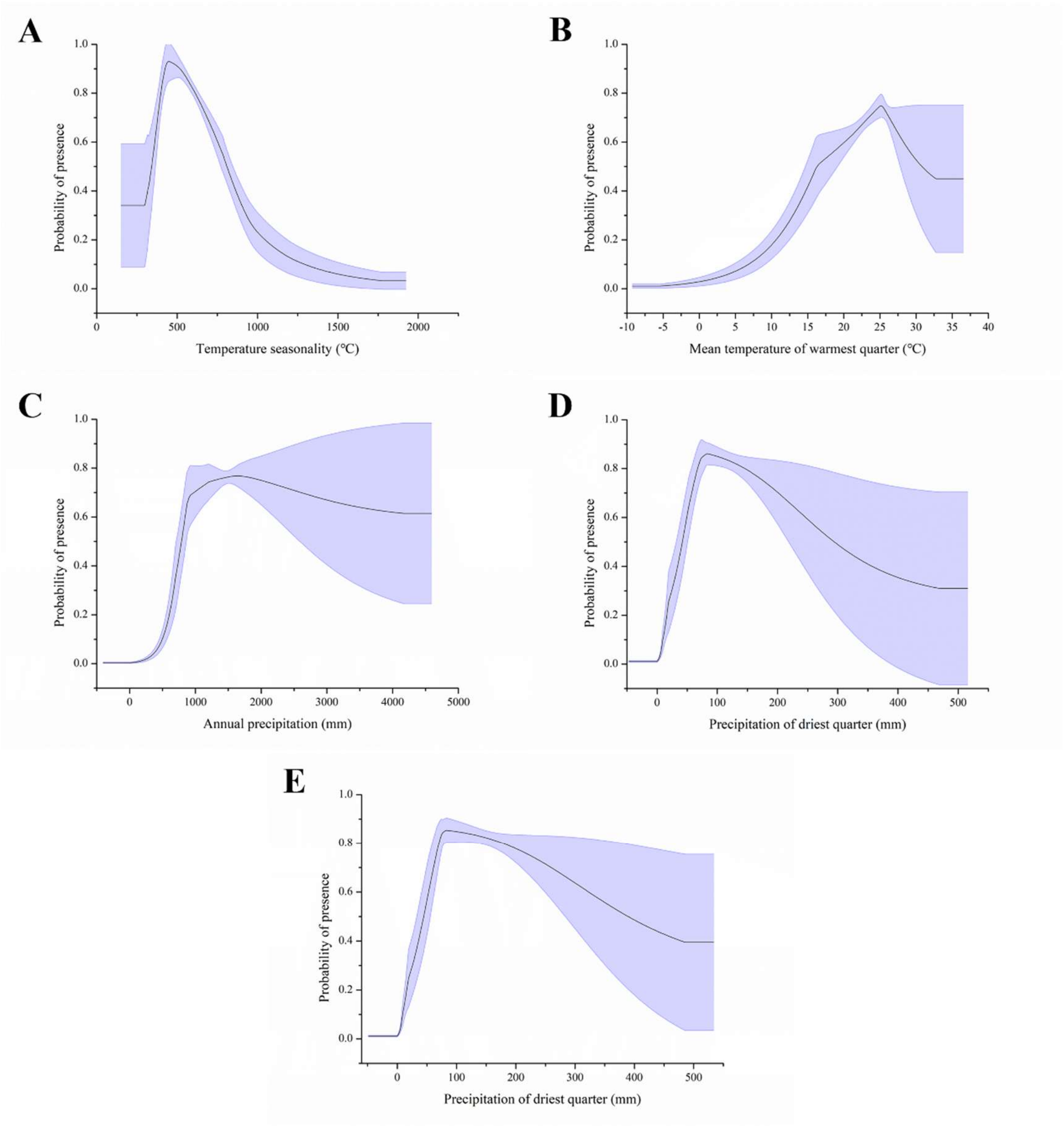

Figure S1. Response curves of existence probability of *L. edodes* distribution model with host plants to Bio4 Temperature seasonality (A), Bio10 Mean temperature of warmest quarter (B), Bio12 Annual precipitation (C), Bio17 Precipitation of driest quarter (D), Bio19 Precipitation of coldest quarter (E)

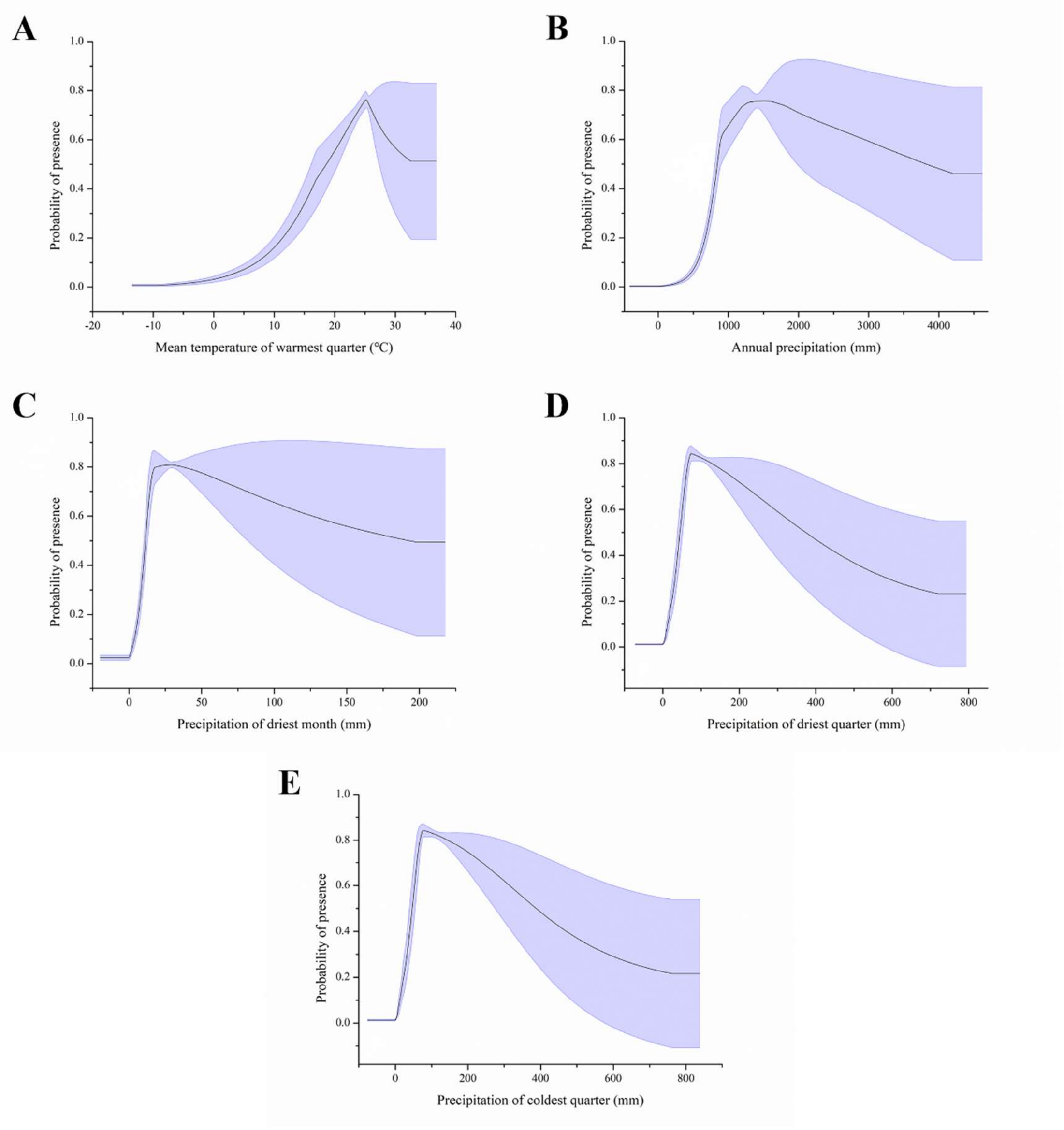

Figure S2. Response curves of existence probability of *L. edodes* distribution model excluding host plants to Bio10 Mean temperature of warmest quarter (A), Bio12 Annual precipitation (B), Bio14 Precipitation of driest month (C), Bio17 Precipitation of driest quarter (D), Bio19 Precipitation of coldest quarter (E)
